# Supplementary material for: A Critical View of the Application of the APEX Software (Aqueous Photochemistry of Environmentally-Occurring Xenobiotics) to Predict Photoreaction Kinetics in Surface Freshwaters
Source: Molecules. 2019 Dec 18;25(1):9. doi: 10.3390/molecules25010009 (PMC7017383; doi:10.3390/molecules25010009)
Supplement: Supplementary file 1 [file molecules-25-00009-s001.zip › 3.2.4_gcc-4.4.0.zip!/3.2.4_gcc-4.4.0/3.2.4_gcc-4.4.0/doc/octave/html/interpreter/Image-Properties.html]

Image Properties - Untitled


Next: Patch Properties,
Previous: Text Properties,
Up: Graphics Object Properties


---

##### 15.2.2.6 Image Properties

`cdata`: The data for the image. Each pixel of the image corresponds to an element of `cdata`. The value of an element of `cdata` specifies the row-index into the colormap of the axes object containing the image. The color value found in the color map for the given index determines the color of the pixel. `xdata` `ydata`: Two-element vectors specifying the range of the x- and y- coordinates for the image.
